# Medicinal Herbal Recommendation for Irritable Bowel Syndrome in Medieval Persian Medicine

**Published:** 2019-11

**Authors:** Farshad AMINI-BEHBAHANI, Mohsen BAHRAMI, Bagher MINAEI, Behzad EFTEKHAR, Majid DADMEHR

**Affiliations:** 1. Research Institute for Islamic and Complementary Medicine, Iran University of Medical Sciences, Tehran, Iran; 2. School of Persian Medicine, Iran University of Medical Sciences, Tehran, Iran; 3. Department of Histology, School of Medicine, Tehran University of Medical Sciences, Tehran, Iran; 4. Department of Neurosurgery, Nepean Hospital, University of Sydney, New South Wales, Australia

## Dear Editor-in-Chief

Irritable bowel syndrome (IBS) is one of the most common functional gastrointestinal disorder characterized by chronic abdominal pain associated with bowel habit alteration (1). Currently, pharmacological treatment strategies can only provide limited symptomatic relief, therefore, many patients are not completely treated and do not have a proper quality of life, there may also be drug side effects, these have resulted in increasing the tendency to use of complementary and alternative medicines (2).

Although, the term that is the same as IBS cannot be found as a distinct disease in the ancient Persian medicine (PM) manuscripts, considering signs and symptoms of IBS in current diagnostic criteria, the clinical manifestations similar to IBS have been mentioned in the PM sources. PM scientists have described some types of disorders with the relationship of “*Maraqq*” and called them Maraqq-related disorders (3–5). Symptoms of IBS have been described under the topic of “Maraqq-related disorders” and “*Qulanj*” disease (3–5).

In some historical medical textbooks, “*Maraqq*” is a membranous structure in the abdominal region (3–5). It is described as a parietal peritoneum (external layer) (3,5) and a fascia lining the digestive tract (stomach, liver, spleen, intestine and etc.) (4,5). Moreover, all of the abdominal wall layers (skin, muscles and parietal peritoneum) are suggested as the “*Maraqq*” (3,6).

This structure naturally plays an important role in excretory functions of the body, some of them include contribution to peristalsis with the help of abdominal muscles, uterine contractions, urination and exodus of bloating (3).

Some disturbances in the “*Maraqq*” can cause the patients have Maraqq-related symptoms and signs. These clinical manifestations include: pain, burning and distention on the stomach and the “*Maraqq*”, pain between two shoulders, drooling [water brash], sour eructation, abdominal flatulence, loose stool (3–5), borborygmus, heaviness in the “*Maraqq*”, nausea, dyspepsia (3,4), slimming (4,5), constipation (4) and polyphagia (5). Moreover, weakness of abdominal wall muscles “*Maraqq”* resulted in impairment in exodus of bloating and feces, also patients experience abdominal pain (colic) and constipation (7).

The treatment plans suggested for Maraqq-related diseases consist of:

1. Nutritional advice:
  Some of the nutritional advice to help treating these patients include use of apple, barley water, chicken, egg, lettuce, milk, spinach and whey “*Maoljobon*” (3–5).
2. Drug therapy:
  Some herbs suggested for treating this disease are listed in Table 1 (3–5).
3. Manual intervention
  *- *Use of liniment “*Tila*” and plaster “*Dhimad*” form of drugs on the “*Maraqq*” [abdomen] for eliminating flatulence (4).
  *-* A wrapped warm wheat bran and salt in a cloth and put it on the “*Maraqq*” [abdomen] (4,5).
  *-* Rubbing and anointing rose, mastic and hyacinth oil on the “*Maraqq*” [abdomen] or stomach (4,5).
  *-* Use of dry and wet cupping therapy on the “*Maraqq*” [abdomen] for reducing flatulence (3,4).

**Table 1:** Some natural remedies suggested for Maraqq-related disorders in PM sources

***Scientific name***	***Common name ***	***Persian medicine name***	***Temperament***
*Matricaria chamomilla*	Chamomile	*Babonaj *	Hot & dry
*Viola odorata*	Sweet Violet	*Banafsaj *	Cold & moist
*Tamarindus indica*	Tamarind	*Tamr-e Hendi *	Cold & dry
*Juniperus sabina*	Savin	*Abhol *	Hot & dry
*Cichorium intybus*	Chicory	*Hendeba *	Cold & moist
*Cucurbita Pepo*	Pumpkin	*Qar’ *	Cold & moist
*Coriandrum sativum*	Coriander	*Kozborah *	Cold & dry
*Cassia fistula*	Golden shower tree	*Khiarshanbar *	Hot & moist
*Rosa damascena*	Damask rose	*Vard *	Cold & dry
*Pistacia lentiscus*	Mastic	*Mastaki *	Hot & dry
*Melilotus officinalis*	Sweet clover	*Eklilolmalek *	Hot & dry
*Anethum graveolens*	Dill	*Shebat *	Hot & dry

Nowadays, IBS is definitely diagnosed according to the newest version of Rome diagnostic criteria (Rome IV). In the current diagnostic criteria, abdominal pain is a main clinical feature in these patients, recurrent abdominal pain should be present at least 1 day per week in the last 3 months, moreover, alteration in bowel habits is considered as a consistent clinical manifestation in IBS (8,9). Based on stool form alone, IBS is mainly classified into four categories (9).

Current epidemiological studies show that IBS can be more than abdominal pain and bowel habit abnormalities, moreover, pain or burning in the stomach, flatulence (8), abdominal distention and bloating are common complaint too (9). Furthermore, recent findings suggest a significant overlap between functional dyspepsia and irritable bowel syndrome; upper gastrointestinal symptoms such as dyspepsia, heartburn, belching and nausea were found in IBS (1).
